# Indirect Manganese Removal by* Stenotrophomonas *sp. and* Lysinibacillus *sp. Isolated from Brazilian Mine Water

**DOI:** 10.1155/2015/925972

**Published:** 2015-12-01

**Authors:** Natália Rocha Barboza, Soraya Sander Amorim, Pricila Almeida Santos, Flávia Donária Reis, Mônica Mendes Cordeiro, Renata Guerra-Sá, Versiane Albis Leão

**Affiliations:** ^1^Laboratório de Bioquímica e Biologia Molecular, Departamento de Ciências Biológicas (DECBI) and Instituto de Ciências Exatas e Biológica (ICEB), Universidade Federal de Ouro Preto, 35400-000 Ouro Preto, MG, Brazil; ^2^Laboratório de Bio&Hidrometalurgia, Departamento de Engenharia Metalúrgica e de Materiais (DEMET), Escola de Minas, Universidade Federal de Ouro Preto, 35400-000 Ouro Preto, MG, Brazil; ^3^Faculdade Unileste, 35160-215 Ipatinga, MG, Brazil

## Abstract

Manganese is a contaminant in the wastewaters produced by Brazilian mining operations, and the removal of the metal is notoriously difficult because of the high stability of the Mn(II) ion in aqueous solutions. To explore a biological approach for removing excessive amounts of aqueous Mn(II), we investigated the potential of Mn(II) oxidation by both consortium and bacterial isolates from a Brazilian manganese mine. A bacterial consortium was able to remove 99.7% of the Mn(II). A phylogenetic analysis of isolates demonstrated that the predominant microorganisms were members of* Stenotrophomonas*,* Bacillus*, and* Lysinibacillus* genera. Mn(II) removal rates between 58.5% and 70.9% were observed for* Bacillus* sp. and* Stenotrophomonas* sp. while the* Lysinibacillus* isolate 13P removes 82.7%. The catalytic oxidation of Mn(II) mediated by multicopper oxidase was not properly detected; however, in all of the experiments, a significant increase in the pH of the culture medium was detected. No aggregates inside the cells grown for a week were found by electronic microscopy. Nevertheless, an energy-dispersive X-ray spectroscopy of the isolates revealed the presence of manganese in* Stenotrophomonas* sp. and* Lysinibacillus* sp. grown in K medium. These results suggest that members of* Stenotrophomonas* and* Lysinibacillus* genera were able to remove Mn(II) by a nonenzymatic pathway.

## 1. Introduction

Manganese is a common contaminant in many mine waters, groundwater, and freshwaters worldwide [1–8]. Brazil is one of the largest producers of manganese ore, and the state of Minas Gerais has the largest reserves of this metal [9]. Unfortunately, mining activities may result in the dissolution of manganese-containing minerals and lead to the contamination of freshwater with the element. Because its solubility is high, manganese concentrations of up to 100 mg·L^−1^ can be found in some Brazilian mine waters, and this concentration is much greater than the limit that was established by the Brazilian legislation for industrial effluents (i.e., 1.0 mg·L^−1^) [10].

Typically, Mn(II) ions are chemically removed from effluents by oxidation to MnO_2_, adsorption, or precipitation as a carbonate [7, 11–14]. However, chemical processes are often resource intensive because multiple steps are required. Furthermore, such processes may present low efficiencies and produce secondary pollution, such as toxic byproducts [15–17].

Manganese removal using biological processes could be an alternative to chemical routes. The role of microbial activity in the remediation of manganese-contaminated waters has been frequently documented [1, 2, 5, 18–20]. In addition to biosorption, the bacteria-mediated precipitation of MnO_2_ has been proposed. Manganese-oxidizing microorganisms are widespread in nature and are phylogenetically diverse [16, 21–23]. Although several bacteria promote manganese bioremediation, only few species have been extensively studied (i.e.,* Pseudomonas*,* Bacillus*,* Leptothrix*, and* Pedomicrobium*) [5, 24–31]. Furthermore, few studies have identified or tested the ability of local bacterial strains to remove the element despite high Mn(II) concentrations in some mine waters and groundwater in tropical countries. Therefore, the goals of the current study are to (i) enrich a bacterial consortium from mine waters with high Mn(II) concentrations and (ii) isolate and identify the bacterial strains from the enrichment, while assessing their manganese removal ability. This knowledge would be useful for the design of new more efficient strategies for treating manganese-laden wastewaters.

## 2. Material and Methods

### 2.1. Sample Sites

A water sample was collected from a manganese mine in the Iron Quadrangle region (Minas Gerais, Brazil). The mine water was stored in plastic containers at room temperature until laboratory processing. A sample of the mine water was selected and analyzed for metal content by inductively coupled plasma optical emission spectrometry (ICP-OES; Varian 725).

### 2.2. Enrichment

A subsample comprising one liter of mine water was filtered through a 0.22 *μ*m membrane (Millex, Millipore). The membrane was transferred to Falcon tubes with 10 mL of K medium (0.15 g·L^−1^ MnSO_4_·H_2_O, 2 g·L^−1^ peptone, 0.5 g·L^−1^ yeast extract, and 10 mM HEPES buffer, pH 7.5). Cultures were incubated at 30°C on an orbital shaker (150 min^−1^) for 24 h. Subsequently, the cultures were transferred to 250-mL flasks containing 90 mL of K medium and incubated as described above. The obtained consortium was called CL throughout this study. After 24 h, 50% (w/v) glycerol was added, and aliquots of 1 mL were stored in Eppendorf tubes at −80°C until the experiments were conducted.

### 2.3. Isolation Procedures

Pure strains were isolated from the enriched CL consortium by plating on 1.5% (w/v) agar-solidified K medium. The agar plates were amended with 50 mg·L^−1^ Mn(II) ion after autoclave sterilization. The cultures were incubated at 30°C until colonies formed and/or the culture medium containing Mn(II) turned brownish. The colonies, which had changed color and/or changed the media color, were selected, and bacterial isolates were inoculated into K medium without Mn(II). The isolates were preserved at −80°C in 50% (w/v) glycerol.

### 2.4. Manganese Removal Experiments

Batchwise manganese removal experiments at 50 mg·L^−1^ Mn(II) ion concentrations in a 2-week-long period were performed with the CL consortium. These experiments were accomplished in a small-scale 3-L bioreactor (BioFlo 110 Fermentor/Bioreactor, New Brunswick Scientific) at a one-liter final volume. Prior to the bioreactor experiment, 1 mL of the CL consortium preserved at −80°C was cultured in 10 mL of K medium (at 30°C) on an orbital shaker (150 min^−1^) for 24 h (i.e., a preinoculation period). Subsequently, the preinoculum was transferred to 250-mL flasks containing 90 mL of K medium and incubated under the same conditions as described above. Thereafter, 100 mL of the culture (approximately 0.1 optical density at 600 nm) was transferred to a bioreactor containing 900 mL of K medium at pH 7.5 in the presence of 50 mg·L^−1^ Mn(II) and maintained at 30°C under constant stirring (150 min^−1^) for two weeks. Aliquots were periodically collected for manganese concentration, pH, and bacterial growth measurements (by counting cells in a Neubauer chamber). The experiments were performed in duplicate. Concurrently, a similar bioreactor was operated as a control in which the bacterial growth was inhibited by a Nipagin (0.14% w/v)-Nipazol (0.1% w/v) addition.

Manganese removal by the isolates was performed in flasks at final volumes of 100 mL. The preinoculum was prepared as described above for the CL consortium. Subsequently, 10-mL aliquots of the preinoculum were transferred to 250-mL flasks containing 90 mL of K medium that was supplemented with 50 mg·L^−1^ Mn(II) at an initial pH of 7.5. The flasks were maintained at 30°C on an orbital shaker (150 min^−1^) for seven days. Aliquots were collected periodically, and the same analyses that were performed for the consortium CL were performed for the isolates (i.e., manganese concentration, pH, and bacterial growth measurements using the optical density method in a Hitachi 2800 A spectrophotometer). In the control flasks under the same conditions, bacterial growth was inhibited by adding Nipagin (0.14% w/v)-Nipazol (0.1% w/v). The experiments were performed in triplicate.

For manganese analysis, approximately 4-mL aliquots of culture were centrifuged for 15 min at 14,681 ×g and filtered through a 0.22-*μ*m membrane. The filtrate was collected, diluted 10 or 25 times in distilled water, and acidified with HCl (1 : 1) solution. The dilutions were analyzed by ICP-OES. The amount of manganese that was removed was measured by the decay of the element in the culture medium.

We also evaluated the culture growth on solid K media containing different Mn(II) ion concentrations (140 mg·L^−1^, 300 mg·L^−1^, 600 mg·L^−1^, and 1200 mg·L^−1^). Plates were incubated at 30°C for two weeks, after which dark brownish colonies or the browning of the culture medium was observed.

### 2.5. Mn(II) Oxidation Assays

Abiotic manganese oxidation was confirmed in control experiments that were performed at different pH values (from 7.5 to 9.0 in 0.5 steps). Samples of 0.1 mL of the K medium that were amended with 50 mg·L^−1^ Mn(II) and submitted to the same conditions of the experiments with the inoculum were mixed with 0.5 mL of 0.04% Leucoberbelin blue I (LBB, Sigma-Aldrich, USA) in 45 mM acetic acid. As a negative control, K media without 50 mg·L^−1^ Mn(II) or with manganese carbonate (MnCO_3_) solutions were also tested as described above. For a positive control, K medium with manganese oxide (MnO_2_) was used.

### 2.6. Mn(II) Oxidation by Cell-Free Filtrate

The cell-free filtrates were prepared by growing the isolates on K medium for four days. The cultures were then centrifuged at 7,155 ×g for 20 min, the supernatant was filtered through a 0.22 *μ*m pore size membrane filter, and the resulting solution was named the cell-free filtrate. The cell-free filtrate was split in two flasks, one of which received proteinase K (100 *μ*g·mL^−1^, Promega). These flasks (with proteinase K) were incubated at 37°C for three hours before the addition of Mn(II). Subsequently, approximately 30 mg·L^−1^ Mn(II) was added to all of the flasks (i.e., with or without proteinase K), which were maintained at 37°C and 150 min^−1^ for three days. Samples were collected at 0 h, 12 h, 24 h, 36 h, 48 h, 60 h, and 72 hours, and the manganese concentrations were measured by ICP-OES to determine the removal efficiencies. The presence of oxidized manganese species was monitored by the addition of Leucoberbelin blue dye (LBB, Sigma-Aldrich, USA) to the samples.

### 2.7. Multicopper Oxidase Activity

To determine whether multicopper oxidases were involved in manganese oxidation by the isolates, the strains were grown in agar-solidified K medium (1.5%, w/v) that was supplemented with 50 mg·L^−1^ Mn(II) and 2,2′-azino-bis(3-ethylbenzthiazoline-6-sulfonic acid) (ABTS, Sigma-Aldrich, USA). The Petri dishes were incubated at 30°C for up to 30 days, and the multicopper oxidase activity was determined by the development of a blue color in the culture medium [32].

### 2.8. DNA Extraction and DGGE-PCR Analysis of CL Consortium

A 100-mL aliquot of the CL consortium from the manganese removal experiment was centrifuged at 7,155 ×g for 20 min. The pellets were washed three times with sterile PBS (8.18 g·L^−1^ NaCl, 1.98 g·L^−1^ NaHPO_4_·7H_2_O, and 0.36 g·L^−1^ NaHPO_4_·H_2_O, pH 7.2). Then, the pellets were resuspended in 2 mL of sterile PBS, transferred to Eppendorf tubes, and centrifuged at 14,681 ×g for five minutes. DNA was extracted from the pellets using a Wizard Genomic kit (Promega) following the manufacturer's protocol and stored at 4°C prior to use. For the DNAg extraction from isolates, samples were cultured in the absence of Mn(II) ions overnight at 30°C on an orbital shaker (150 min^−1^). Then, the DNAg extraction was performed as described above.

The bacterial communities in consortia from the manganese removal experiment were analyzed by denaturing gradient gel electrophoresis (DGGE). The DGGE primers that were used were 968F/1392R [33], 357F/518R, and 357/907R (968F: 5′-AACGCAAGAACCTTAC-3′; 1392R: 5′-ACGGGCGGTGTGTAC-3′; 357F: 5′-CCTACGGGAGGCAGCAG-3′; 518R: 5′-ATTACCGCGGCTGCTGG-3′; 907R: 5′-CCGTCAATTCMTTTRAGTTT-3′) [34]. The primers 968F and 357F contained a CG clamp 5′-CGCCCGGGGCGCGCCCCGGGCGGGGCGGGGGCACGGGGGG-3′ that was attached to the 5′ end. PCR amplifications were performed in 50-*μ*L reaction mixtures containing 1x Taq Buffer, 1.5 mM MgCl_2_, 0.2 mM dNTP mix, 0.2 mM primers mix, 2.5 U of Taq DNA polymerase (Thermo Scientific* Taq* DNA Polymerase, Fermentas), and 10 ng of DNA template.

For primers 968F/1392R, a PCR program was performed with an initial denaturation step (94°C, 5 min) followed by 35 cycles of denaturation (94°C, 45 s), annealing (63°C, 1 min), and extension (72°C, 2 min) and a single final extension step (72°C, 20 min). For primers 357F/518R and 357F/907R, the PCR thermocycling regime was performed with an initial denaturation step (94°C, 5 min), followed by 35 cycles of denaturation (94°C, 1 min), annealing (49°C, 1 min), and extension (72°C, 1 min) and a single final extension step (72°C, 20 min). The PCR products were visualized by 1.2% agarose gel electrophoresis.

Subsequently, 20 *μ*L of PCR product was subjected to DGGE analysis as described by Muyzer et al. [35] following the manufacturer's protocol for the CBS System Scientific EPS-30 II model DGGE-2401. For the DGGE analysis of the CL consortium, a denaturing gradient ranging from 20 to 60% (primers pairs 968F/1392R and 357F/907R) or 40 to 60% (primers pair 357F/518R) was used (a 100% denaturant was defined as 7 M urea and 40% formamide). Electrophoresis was performed in a TAE buffer at 60°C and 100 volts for 15.5 h. The gels were stained with ethidium bromide and visualized under UV light.

### 2.9. 16S rRNA Gene Amplification, Sequencing, and Phylogenetic Analyses

DNAg was extracted from the isolated pure cultures. PCR was performed to amplify a region of the 16S rRNA gene from DNAg extracts using primers 968F/1392R as described above (DNA Extraction and DGGE-PCR Analysis of CL Consortium). The PCR products were purified using standard protocols [36] and sequenced on a 3500 Genetic Analyzer (Applied Biosystems). The quality of the 16S rRNA gene sequences was checked using the multiple alignment CLUSTALW software package [37]. A phylogenetic analysis was performed using the neighbor-joining method and the Jones-Taylor-Thornton model [38]. The tree topologies were evaluated by performing a bootstrap analysis with 1,000 replicates. Phylogenetic analyses were conducted using the Figtree 1.4 software.

### 2.10. Nucleotide Sequence Accession Numbers

The partial sequence of the 16S rRNA gene sequence of* Bacillus* sp.,* Lysinibacillus*, and* Stenotrophomonas* was deposited in the GenBank under the accession number from KT962904.1 to KT962906.1 and from KT970681.1 to KT970697.1.

### 2.11. Scanning Electron Microscopy (SEM), Transmission Electron Microscopy (TEM)/Energy-Dispersive X-Ray Spectroscopy (EDX) Analyses

The isolates with Mn(II) removal activity were cultured in liquid K medium containing 50 mg·L^−1^ Mn(II) for 7 days as previously described. Subsequently, the suspensions were centrifuged and prepared for SEM/TEM analyses. SEM/EDX assays were performed using a JSM-6360/LV and FEG-Quanta 200 FEI, while a TEM/EDX analysis was performed with a Tecnai G2-12-SpiritBiotwin FEI-120 kV and a Tecnai G2-20-SuperTwin FEI-200 kV. Experiments and analyses involving electron microscopy were performed in the Center of Microscopy at the Universidade Federal de Minas Gerais, Belo Horizonte, MG, Brazil.

## 3. Results

### 3.1. Manganese Removal by the Bacterial Consortium

**Figure 1:**
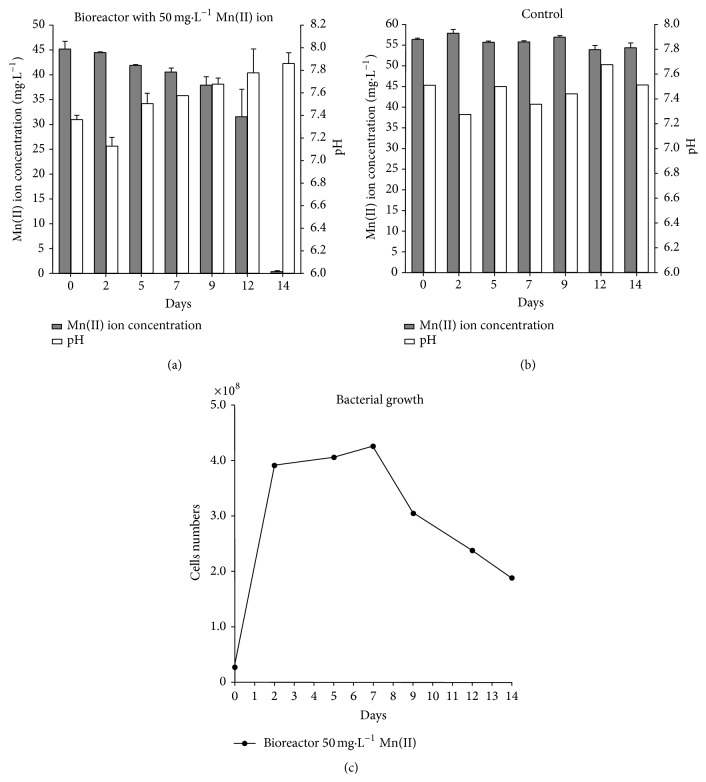
Growth and pH variation during Mn(II) ion removal by CL consortium in a 2-week period. The CL consortium was inoculated in K medium and was maintained in a bioreactor at 30°C and under constant stirring (150 min^−1^).

The analyses of the mine water under study showed manganese as one of the major constituents (140 mg·L^−1^) at pH 6.5 [14]. Using the K medium, the CL consortium that was enriched from this mine water was tested for its Mn(II) removal ability as described in Section 2. In these experiments, the initial metal concentration was reduced to 50 mg·L^−1^ to minimize the manganese removal by air oxidation, which is catalyzed at higher pH values [39]. The amount of Mn(II) that was removed by the consortium and the profiles of both pH and bacterial growth during the experiment are shown in Figure 1. The concentration of Mn(II) continuously decreased over time in the presence of the CL consortium with a removal efficiency of 99.7%, and the manganese residual concentration was 0.2 mg·L^−1^, whereas the pH varied between 7.36 (initially) and 7.86 at the end of the experiment (Figure 1(a)).

Unlike the inoculated tests, the pH values did not increase in the control experiment and remained around 7.4, whereas no significant manganese removal (3.44%, after 14 days) was observed (Figure 1(b)). This outcome suggests that the pH increase that is shown in Figure 1(a) was promoted by bacterial growth.

The bacterial counts peaked (4.0 × 10^8^ cells/mL) within two days when the Mn(II) removal started (Figures 1(a) and 1(c)). The bacterial population was stable until the seventh day, and thereafter, a slight decrease in cell counts was observed, reaching 2.5 × 10^8^ cells/mL on the 12th day. At this time, we observed greater manganese removal (Figure 1(a)).

### 3.2. Phylogenetic Analysis of the Isolates from the CL Consortium

**Figure 2:**
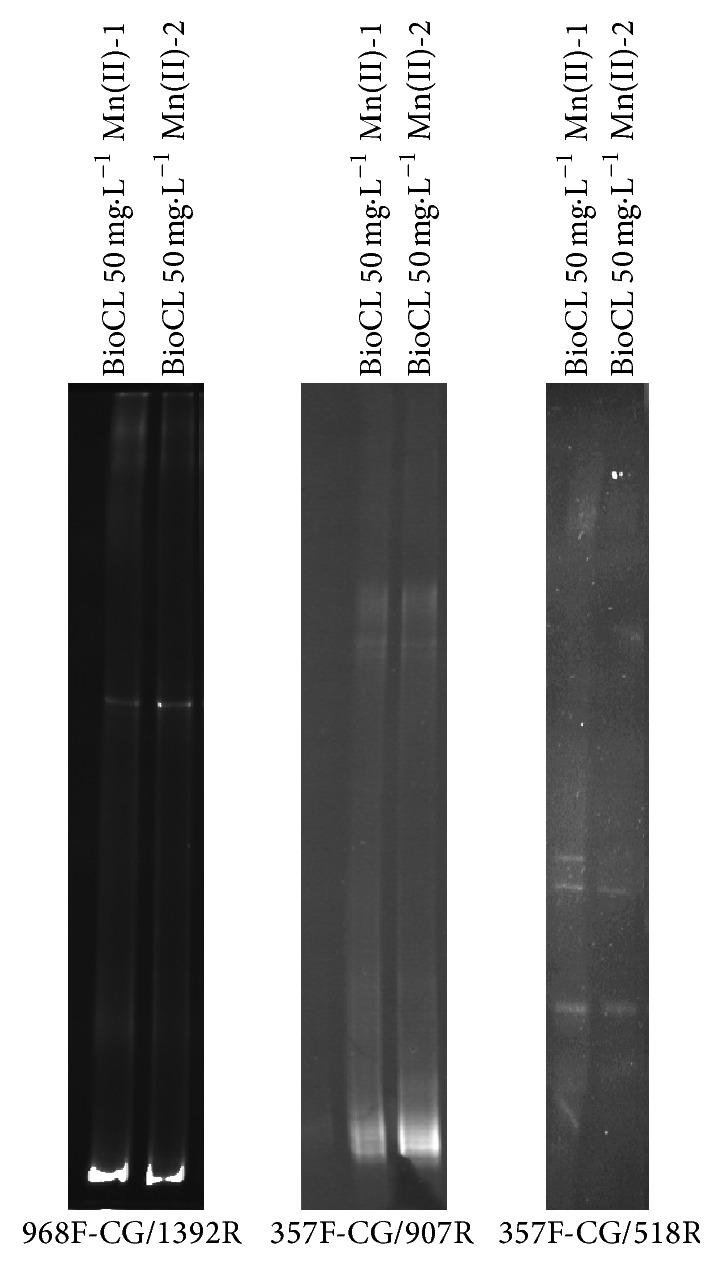
CL consortium molecular characterization. DGGE analysis of the bacterial 16S rRNA gene as amplified from the total community DNA of the CL consortium.

At the end of the 14-day Mn-removal experiment, the microorganisms were recovered by centrifugation, and the collected pellets were used for DNAg extraction and DGGE-PCR analysis. The DGGE profile suggests low microbial diversity because even when using several sets of primers to amplify different regions of the 16S rRNA, few amplicons were obtained (Figure 2).

**Figure 3:**
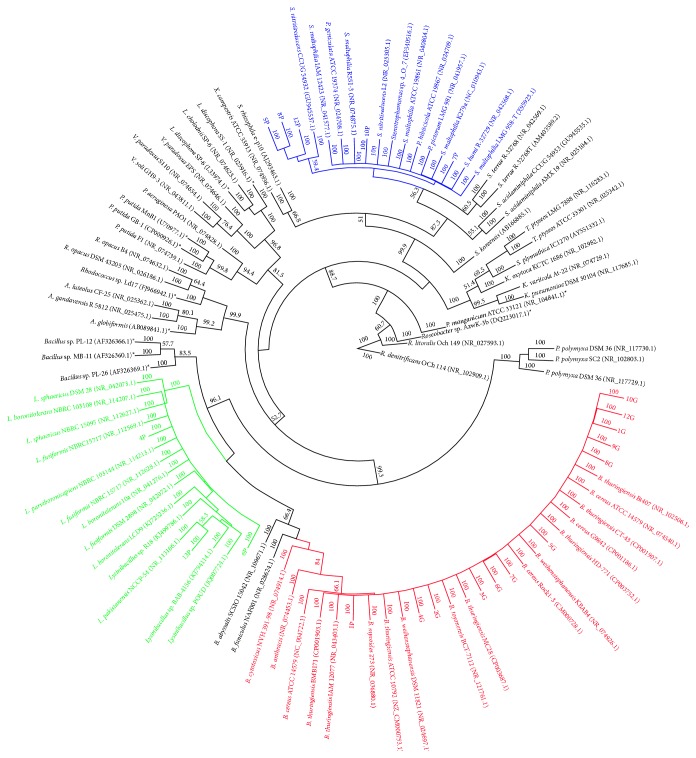
Phylogenetic relationship between the partial 16S rRNA gene sequences from CL consortium isolates and their closest GenBank sequences with the 16S rRNA gene from previously reported known Mn(II)-oxidizing bacterial strains (labeled with *∗*). The GenBank accession numbers of these sequences are shown in brackets. Bootstrap values ≥ 50% with 1,000 replicates are indicated at the branch points.

Bacterial species identification after sequencing of the 16S rDNA fragments from the DGGE gel was not conclusive, and an isolation procedure was used to determine the diversity of the CL consortium, which resulted in 20 isolates (data not shown). To identify the genera of the Mn(II)-oxidizing bacteria that were isolated in this study, we sequenced the 968F/1392R 16S rRNA amplicons and used them for the phylogenetic study. As shown in Figure 3, 15 isolates belonged to the Firmicutes phylum, with another five belonging to the Gammaproteobacteria phylum. Among the Firmicutes isolates, 12 clustered with species from the* Bacillus cereus* group, and the other three isolates were phylogenetically closer to the genus* Lysinibacillus* (4P, 6P, and 13P). The five isolates belonging to the phylum Gammaproteobacteria were clustered with genus* Stenotrophomonas*. We also performed a biochemical test using the Bactray I, II, and III system to identify the species (data not shown). The results corroborated the phylogenetic analyses.

### 3.3. Manganese Removal by the Isolates

Initially, two representatives of each identified genus (*Bacillus*,* Stenotrophomonas*, and* Lysinibacillus*) were chosen for growth in solid K medium containing different Mn(II) ion concentrations (140 mg·L^−1^, 300 mg·L^−1^, 600 mg·L^−1^, and 1200 mg·L^−1^) to determine whether these genera were capable of Mn(II) removal as indicated by the color of the colony and/or of the medium. These experiments also showed the tolerance of isolates to manganese. The six isolates were able to be cultured in the presence of 1200 mg·L^−1^ Mn(II) (Figure S1, in Supplementary Material available online at http://dx.doi.org/10.1155/2015/925972); however, growth at this concentration was impaired. Moreover, colonies of* Lysinibacillus* sp. isolates (6P and 13P) showed a brownish color after one week of growth in 140 mg·L^−1^, 300 mg·L^−1^, and 600 mg·L^−1^ Mn(II), suggesting the capacity of Mn(II) oxidation to either Mn(III) or Mn(IV), whereas the remaining isolates only made the culture medium color darker (Figure S1).

**Figure 4:**
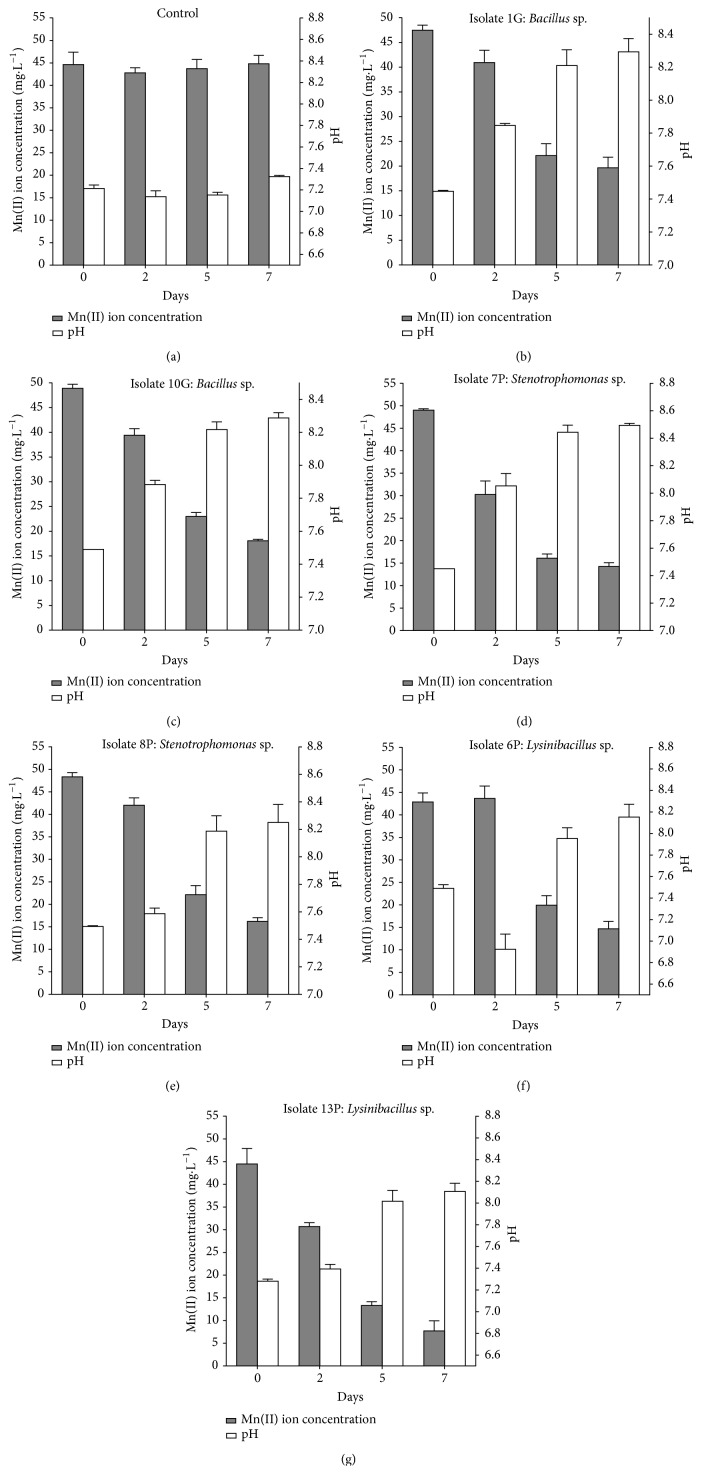
Time course of Mn(II) removal and the pH level of six isolates from the CL consortium. The cells were grown in K medium for one week. The concentration of Mn(II) was determined according to procedures that were described in Section 2.

The analysis of the manganese concentration in liquid medium is shown in Figure 4. The* Bacillus* isolates (1G and 10G) showed no statistical differences in manganese removal capacities (Figures 4(b) and 4(c)). The 1G isolates removed 27.81 mg·L^−1^ of the Mn(II) (58.5%), whereas the 10G isolates removed 30.83 mg·L^−1^ of the Mn(II) (63.0%). Among the* Stenotrophomonas* sp. isolates (7P and 8P), the same was observed (Figures 4(d) and 4(e)). The 7P isolate removed 34.78 mg·L^−1^ of the Mn(II) (70.9%) from culture medium, and the 8P isolate removed 32.12 mg·L^−1^ of the Mn(II) (66.4%). For* Lysinibacillus* sp. isolates, there was a difference in the manganese removal ability: 6P removed only 28.17 mg·L^−1^ of the Mn(II) (65.58%), whereas the 13P isolate removed 36.76 mg·L^−1^ of Mn(II) during the experiments (82.7%). In the control test (Figure 4(a)), no manganese removal within seven days of the experiments was observed.

The solution pH increased to approximately 8.3 in the experiments with* Bacillus* sp. isolates (1G and 10G, Figures 4(b) and 4(c)) and* Stenotrophomonas* sp. isolate 8P (Figure 4(e)), whereas it reached 8.5 for* Stenotrophomonas* sp. isolate 7P (Figure 4(d)). In the experiments with* Lysinibacillus* sp. isolates (6P and 13P), the pH increased only to 8.1 (Figures 4(f) and 4(g)).

Similar to the experiment with the CL consortium, the medium pH did not increase in the control experiment (Figure 4(a)), which indicates that the pH increase in the biotic experiments was promoted by bacterial growth (Figures 1(a) and 4).

### 3.4. Abiotic Manganese Oxidation in Liquid Medium

**Figure 5:**
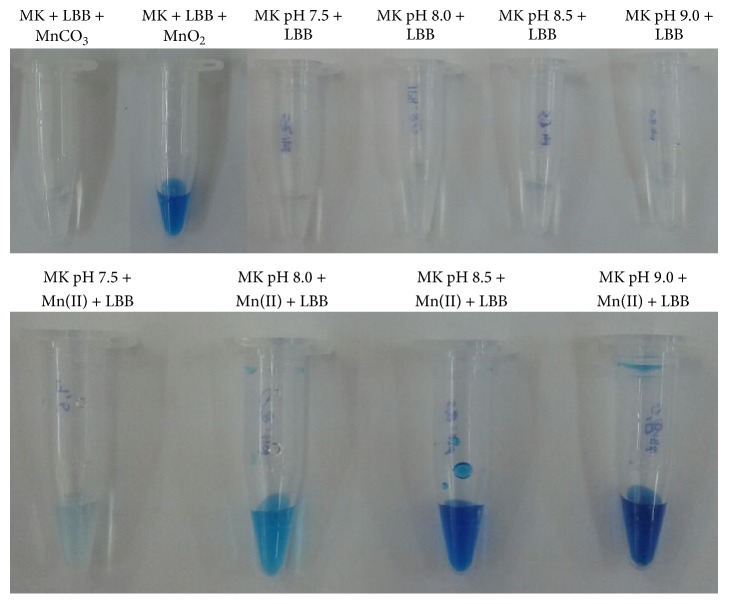
Role of pH in manganese oxidation. K medium that was supplied with or without 50 mg·L^−1^ Mn(II) at different pH values (7.5–9.0) was maintained at 30°C and under constant stirring (150 min^−1^) for seven days. The samples were collected, and 0.04% LBB reagent was added to them. Manganese oxidation was indicated by the sample color turning blue. MK: K medium; LBB: Leucoberbelin blue I reagent; MnCO_3_: manganese carbonate; MnO_2_: manganese oxide.

The role of pH in manganese oxidation was analyzed by submitting K medium that was supplemented with 50 mg·L^−1^ at different pH values under similar conditions to those that were used in experiments of manganese removal by the isolates. Upon LBB addition to 0.1-mL K medium samples, the Mn^IV^O_2_-bearing tube in Figure 5 developed a blue color because of a redox reaction with the dye, whereas the flask containing Mn^II^CO_3_ remained transparent in the presence of LBB. Figure 5 also indicated that the K medium did not contain species that would produce a blue color after mixing with such reagent. Abiotic Mn(II) oxidation can be inferred from the blue color that developed when the Mn(II) solutions were mixed with LBB at increasing pH values; that is, Mn(II) oxidation was catalyzed by an increase of 0.5 units of pH, as indicated by blue color development (Figure 5). Thus, pH played an important role in manganese oxidation promoted by CL consortium and the isolates.

**Figure 6:**
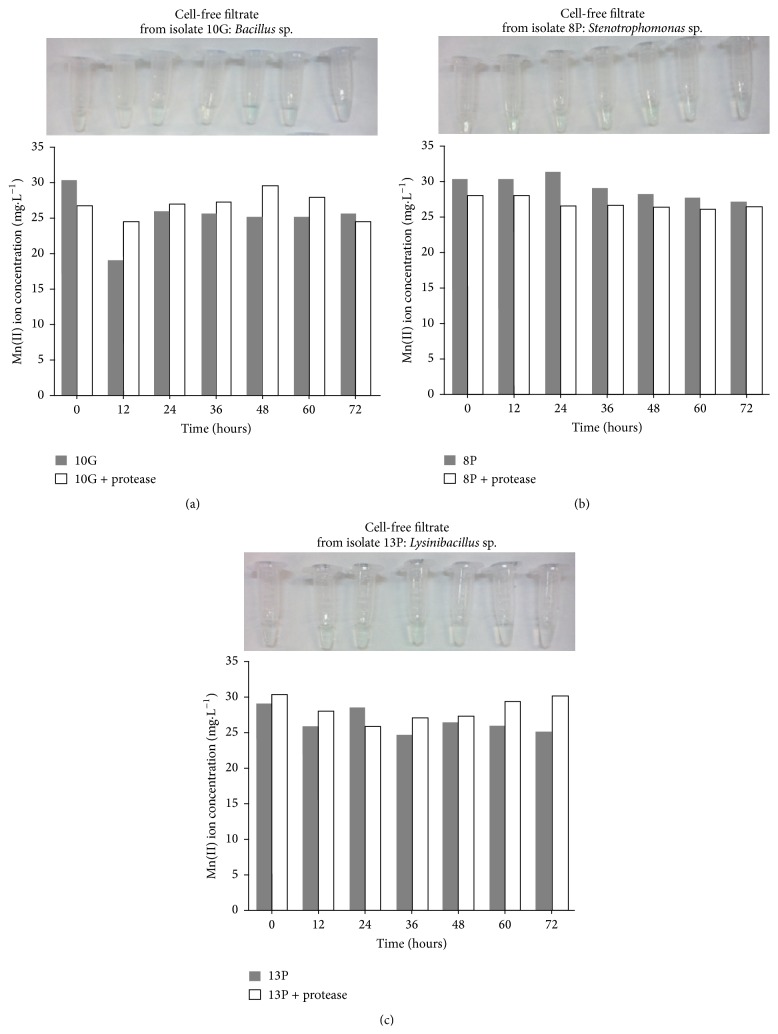
Cell-free filtrate Mn(II) oxidation. The experiments were conducted at 37°C. For certain experiments, 100 *μ*g/mL protease was added to the filtrate to examine the role of proteins in Mn(II) oxidation. The samples were collected periodically, and manganese removal was measured by inductively coupled plasma optical emission spectrometry. The presence of manganese oxides was monitored by the addition of LBB to the samples.

To assess whether bacterial growth would result in the production of extracellular proteins that could oxidize Mn(II), we conducted Mn(II) oxidation assays with cell-free filtrates in either the presence or absence of protease K. We did not observe Mn(II) removal by any cell-free filtrates from isolates 10G (Figure 6(a)), 8P (Figure 6(b)), and 13P (Figure 6(c)) in either presence or absence of protease (Figure 6(a)). Similar results were also observed with the cell-free filtrates from isolates 1G, 7P, and 6P (data not shown). These results indicate that no extracellular proteins were involved in manganese oxidation by the isolates that were selected herein.

### 3.5. Multicopper Oxidase Activity

Manganese oxidation by many bacteria occurs through an enzymatic pathway with multicopper oxidase enzymes (MCO) [29, 40, 41]. Thus, the contribution of MCO to manganese oxidation by isolates 1G, 10G, 7P, 8P, 6P, and 13P was investigated using the ABTS substrate [31, 32]. As a positive outcome, a blue color would develop in the culture medium. As shown in Figure S2, we did not observe the development of a blue color in any enzymatic assays with isolates 10G, 8P, and 13P, although the color of the medium darkened in the presence of Mn(II) ions. The same was observed for isolates 1G, 7P, and 6P (data not shown).

### 3.6. SEM/EDX and TEM/EDX Analyses

**Figure 7:**
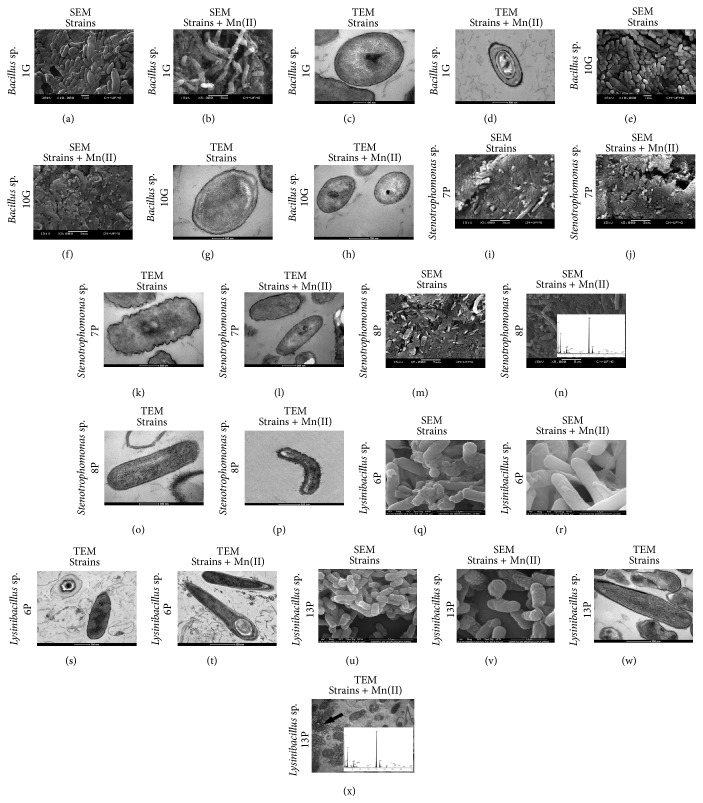
SEM and TEM images of isolates 1G, 10G, 7P, 8P, 6P, and 13P that were cultured on K medium that was supplied with or without 50 mg·L^−1^ Mn(II), EDX spectra of selected areas from the cell surface.

Isolates 1G, 10G, 7P, 8P, 6P, and 13P were cultured in laboratory shaker flasks for seven days and selected for micromorphological responses to the presence of Mn(II) ions. SEM and TEM analyses revealed no aggregates within the cells after seven days of culturing (Figure 7). However, when isolate 13P was grown in the presence of Mn(II) ions, we observed an exopolymer out of the cell that contained manganese, as revealed by EDX spectra (Figure 7(x)). The EDX spectra (Figure 7(n)) also revealed the presence of manganese on the surface of isolate 8P. Furthermore, we could observe spores in culture of* Bacillus* sp. (Figure 7(d)) as expected, as* Bacillus* spp. form spores and, therefore, survive harsh conditions [42]. In addition, a spherical spore was observed in the culture of* Lysinibacillus* sp. (Figure 7(t)). This spore is a defining feature of this bacterium [43].

## 4. Discussion

As stated, we investigated the use of bacteria that were collected from mine waters for potential Mn(II) bioremediation. This analysis was accomplished by enriching and isolating bacterial strains that grow in the presence of high Mn(II) concentrations (up to 1200 mg·L^−1^). Both the consortium and isolated strains removed Mn(II) ions from the culture media.

Specifically, the CL consortium removed 99.7% of Mn(II), which corresponded to a residual metal concentration of 0.2 mg·L^−1^ at the end of the experiment (Figure 1(a)) for an initial concentration in the bioreactor of 45 mg·L^−1^. This residual concentration was lower than the limit that was established by the Brazilian regulations for effluents [10]. Moreover, the final pH increased to 7.86 (Figure 1(a)) with the consortium. A similar pH increase during Mn(II) removal by the isolates was also observed (Figure 4), whereas both negligible Mn(II) removal and no increase in pH were observed in the control experiments (Figure 1(b)). Therefore, the change in solution pH contributed to manganese removal. This result was speculated because pH is one of the major factors affecting manganese oxidation [1, 7, 8, 14, 44]. Furthermore, XANES spectroscopy analysis of the solids that formed during the bioreactor experiments with the CL consortium and in the experiments with the isolates belonging to the* Bacillus* (1G and 10G) and* Stenotrophomonas* (7P and 8P) genera indicated that Mn(II) was precipitated as MnO_2_ (data not shown) as the pH increased. Therefore, we hypothesize herein that bacterial metabolism promoted the increase of pH, which in turn enabled Mn(II) removal by chemical oxidation, as will be further discussed.

Regarding the strains that were isolated in the current study, five were identified as* Stenotrophomonas* sp. (Figure 3). Members of this genus play important ecological and clinical roles and tolerate high concentrations of metals, such as Ag, Cd, and Hg [45]. Although these species can be found in a wide range of environments, for example, in soil, plants, waste gas biofilters, and sewage [46], we showed for the first time that strains of this genus have tolerance to manganese (Figure S1) and promote its removal by the oxidation of Mn(II) ions (Figure 4).

Isolates 4P, 6P, and 13P were identified as* Lysinibacillus* sp., while the other 12 isolates belonged to the* B. cereus* group* sensu lato* (Figure 3). Cerrato et al. [25] isolated* B. cereus* and* Lysinibacillus* sp. strains from a biofilm in drinking water systems and found that* B. cereus* and* L. sphaericus* isolates were capable of some manganese oxidation. Other studies also related* B. cereus* and* L. sphaericus* with Mn(II) removal by either oxidation or adsorption [47, 48], but the mechanism was not discussed by these authors. Furthermore, in such works, the strains removed less Mn(II) and needed more time to promote the manganese removal than did the strains that were isolated in the current study.

SEM/EDX and TEM images (Figures 7(n) and 7(x)) revealed manganese outside the cells (as indicated by an arrow) of isolate 8P (*Stenotrophomonas* sp.) and in an exopolymer-like structure in isolate 13P (*Lysinibacillus* sp.). For the latter, the colonies turned darker in the presence of Mn(II), which may indicate manganese biosorption by this isolate. These observations are consistent with those of other works, which report manganese deposition around the exosporium of* Bacillus* sp. SG-1 and around the sheaths in* Lysinibacillus* sp. [15, 49]. For the latter, no manganese was detected inside the cells. The inability of some manganese-oxidizing microorganisms to accumulate manganese oxides around their cells has been ascribed to the lack of exopolymer production, as for the marine bacteria strain SSW_22_ [15]. In that case, the colonies did not turn brown but caused the browning of Mn(II)-bearing agar. Figure S1 of the current study shows a similar behavior for isolates 1G, 10G, 7P, and 8P, which did not turn the colonies brown. It is likely that these isolates were not able to produce an exopolymer for manganese deposition, making it impossible to find manganese around the cells.

Although few manganese removal studies with* B. cereus* strains have been published so far, the mechanism of manganese oxidation by* Bacillus* SG-1 has been extensively studied, and MCO are involved in this process [50, 51]. Such a manganese oxidation mechanism was also related to other bacteria, such as* Pseudomonas putida* (GB-1 and MnB1) and* L. discophora*, SS-1 [30, 40, 41, 50, 51]. In the current study, no MCO activity was observed during manganese removal by the isolates (Figure S2). Furthermore, we also tested whether some extracellular proteins were involved in manganese oxidation. The results (Figure 6) indicate no participation of the proteins in the cell-free filtrates that were produced from each isolate in manganese oxidation in the current study because no manganese removal was observed with or without proteinase K addition (Figure 6). These results suggest that manganese oxidation by strains of* Bacillus*,* Stenotrophomonas*, and* Lysinibacillus* did not occur by a direct mechanism.

In addition to the enzymatic mechanism involving MCO, there is also an indirect or nonenzymatic pathway for the removal of manganese through the metabolism or growth of microorganisms. This important mechanism appears to have been overlooked in many studies addressing biological manganese removal because pH is a key parameter in Mn(II) oxidation, as previously described. Mn(II) oxidation by oxygen is thermodynamically favorable, but its kinetics are slow. Hydroxyl (OH^−^) ions are among the many catalysts of chemical manganese oxidation, and the rate increases 10 times as the pH is changed from 8.0 to 8.8 [52]. Manganese oxyhydroxides are also catalysts of Mn(II) oxidation, particularly Mn(OH)_2_. When its solubility limit is reached, the precipitated Mn(II)-hydroxide is quickly oxidized to Mn(III) and Mn(IV) [52]. Therefore, chemical manganese oxidation does occur when the pH is increased to values above 8.0 as demonstrated by the results in Figure 6.

Despite many attempts in buffering the experimental conditions using HEPES, the bacterial growth promotes an increase in pH values in the current study (Figures 1 and 5). Therefore, we propose that the biological manganese oxidation by the isolates and the CL consortium followed a nonenzymatic pathway because the final pH attained values in which chemical manganese oxidation predominated. This argument is supported by the fact that we observed Mn(II) oxidation in abiotic experiments (Figure 5). In summary, the presence of manganese-oxidizing bacteria is not required for Mn(II) oxidation provided that the bacteria can increase the solution pH.

## 5. Conclusions

These results extend our knowledge about Mn(II) removal by oxidation. It was demonstrated that mine bacteria are efficient in Mn(II) removal through an indirect pathway by increasing the pH growth medium. The obtained results allow us to conclude the following:

- The CL consortium that was obtained from the mine water was able to remove 99.7% of the manganese that was present in the growth medium, and the residual manganese concentration was 0.2 mg·L^−1^.
- Three different genera of manganese-removing bacteria were isolated from the CL consortium. Most of the isolates belonged to the* Bacillus* sp. genus. The second-most-abundant genus was* Stenotrophomonas*, and bacteria belonging to this genus were related to manganese oxidation for the first time. Three isolates were identified as* Lysinibacillus*.
- The six studied isolates (two representatives for each genus) were able to oxidize manganese and promote manganese removal range from 58.5% to 82.7% in seven days in laboratory-scale reactors.
- We did not detect multicopper oxidase activity, although manganese oxidation at pH values of 8.0 and above was confirmed. We then proposed a nonenzymatic manganese oxidation pathway comprising the modification of the pH growth medium by the isolates.
- The ability of the isolates to increase the pH growth medium could be useful in the treatment of mine waters by producing alkalinity and oxidizing manganese concomitantly, which are key to improving commercial applications.

## Supplementary Material

The data presented here is the gating strategy and representative plots for flow cytometry analysis. Supplementary Figure 1 shows the gating strategy of identifying T and B lymphocytes whereas Supplementary Figure 2 shows the gating strategy for definition of T cell subpopulations. Representative plots for analysis of blood Th cells and Treg are shown in Supplementary Figure 3.
