# Supplementary material for: Indirect Manganese Removal by Stenotrophomonas sp. and Lysinibacillus sp. Isolated from Brazilian Mine Water
Source: Biomed Res Int. 2015 Dec 1;2015:925972. doi: 10.1155/2015/925972 (PMC4678070; doi:10.1155/2015/925972)
Supplement: Supplementary file 1 — The data presented here is the gating strategy and representative plots for flow cytometry analysis. Supplementary Figure 1 shows the gating strategy of identifying T and B lymphocytes whereas Supplementary Figure 2 shows the gating strategy for definition of T cell subpopulations. Representative plots for analysis of blood Th cells and Treg are shown in Supplementary Figure 3. [file 925972.f1.docx]

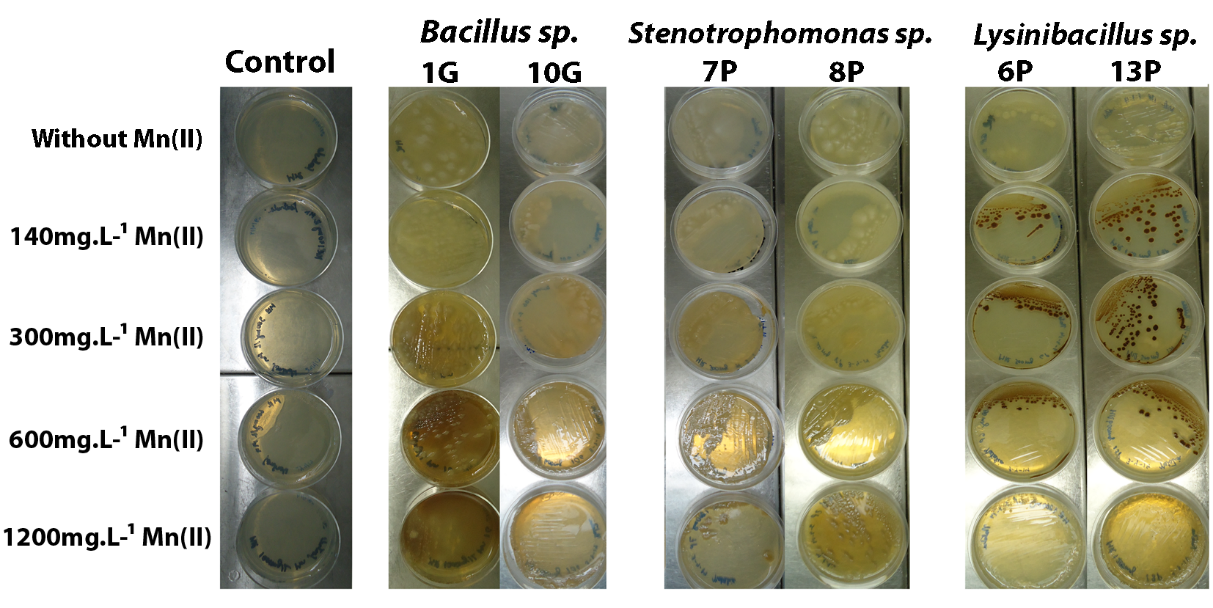


**Figure S1:** Isolates 1G, 10G, 7P, 8P, 6P and 13P were grown on K agar medium with different concentrations of Mn(II) ions. The isolates were cultured at 30°C for two weeks.


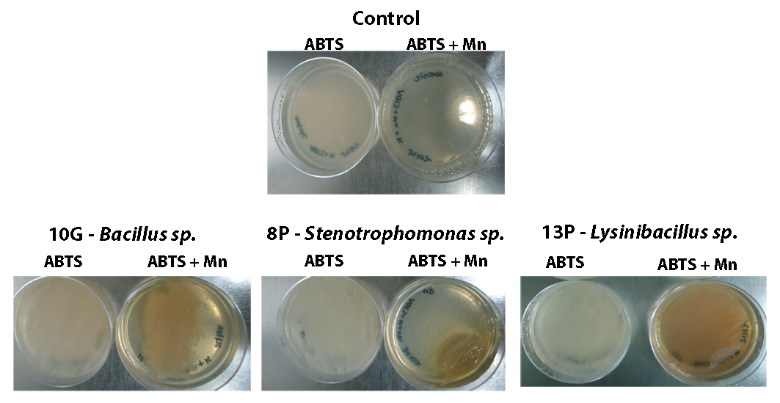


**Figure S2:** Multicopper oxidase activity of the isolates. The strains were grown in agar-solidified K medium with 50 mg.L^-1^ Mn(II) and 0.4 mM ABTS for 4 week to assess the enzymatic activity.
